# Ageism in Storytelling: How Age Bias Influences Perceptions of Story Quality

**DOI:** 10.1093/geroni/igaf122.2198

**Published:** 2025-12-31

**Authors:** Annalise Evans, Nicole Alea

**Affiliations:** University of California, Santa Barbara, Santa Barbara, California, United States; University of California, Santa Barbara, Santa Barbara, California, United States

## Abstract

Ageism permeates many facets of an older adult’s life, even, perhaps, the way that the autobiographical stories they tell are perceived. Older adults are often criticized for going “off-target” or including too many story asides (i.e., tangentially-related information) when telling stories. The paradox is that their stories are also sometimes rated as higher in quality, compared to younger adults. This study aimed to determine if ageism contributes to story quality, depending on the age of the storyteller and how many story asides they used. College-aged participants (N = 123, M = 19.01, 21% male) rated the quality (i.e., entertainment, emotion, memorability, originality, imagery, and engagement) of eight real autobiographical stories that experimentally varied by age of the storyteller (young, old) and amount of story asides (few, many). They were also given an ageism questionnaire. Higher levels of ageism was associated with lower quality ratings for the older adults’ autobiographical stories, regardless of whether those stories were low or high on story asides. Ageism, however, was not associated with the quality of either of the young adults’ stories. Results suggest that the driver of younger adults’ perceptions about the quality of stories may not be the amount of story asides, but bias against older adult storytellers.

